# From statutory mandate to institutional integration: lessons from the national survey of Israel’s healthcare ethics committees

**DOI:** 10.1186/s13584-026-00785-w

**Published:** 2026-10-06

**Authors:** Alona Paz, Maya Peled-Raz, Shlomit Zuckerman, Tatiana Talya Fleishman

**Affiliations:** 1 School of Public Health, Faculty of Social Welfare and Health Studies, University of Haifa, Haifa, Israel; 2 School of Public Health, Gray Faculty of Medicine, Tel Aviv University, Tel Aviv, Israel; 3 Division of General Medicine, Medical Directorate, Ministry of Health, Jerusalem, Israel; 4 The Israeli Nursing Ethics Bureau, Jerusalem, Israel

**Keywords:** Ethics committees, Clinical, Ethics consultation, Health policy, Bioethics education, Patient rights, Israel

## Abstract

**Background:**

Israel’s Patients’ Rights Act (IPRA, 1996) established a unique statutory model for ethics committees (ECs), with legally binding authority, differing from the advisory nature of ECs in most countries. Following the 2015 State Comptroller’s report highlighting widespread under-implementation, the Ministry of Health (MOH) issued a directive in 2018 aimed at standardizing EC structure, procedures, and training. However, the extent of subsequent implementation and operational activity has remained unclear.

**Methods:**

This secondary analysis of the 2022‑2023 MOH national survey examined EC structure, governance, training, and activity in 52 institutions (response rate: 71%), including general, geriatric/rehabilitation, and psychiatric hospitals, MOH district bureaus, health maintenance organizations (HMOs), and the Israel Defense Forces (IDF). Institutional characteristics were merged from MOH administrative databases and compared with available 2015 baseline data. Logistic regression and Generalized Estimating Equation (GEE) models were used to identify factors associated with low EC activity. Low EC activity was defined as a committee that reported both one or fewer (≤ 1) meetings AND one or fewer (≤ 1) applications per year. Conversely, committees reporting more than one meeting or more than one application annually were classified as having high activity for the purpose of the analysis.

**Results:**

The prevalence of statutory ECs was higher in 2022 (89.7%) than in 2015 (70.9%), and the proportion of committees maintaining written protocols was higher (91% vs. 67%), consistent with substantial structural consolidation. However, more than half of institutions were characterized by low EC activity, reporting one or fewer meetings as well as application per year. Low activity was associated with smaller hospital size, geriatric/rehabilitation hospital type, private ownership, lack of trained members, and absence of internal ethics advisors. In contrast, the presence of trained members and a designated contact person was associated with higher EC activity, while advertising alone was not.

**Conclusions:**

Israel’s experience demonstrates that robust statutory mandates and top-down regulatory directives successfully drive formal structural consolidation, yet remain insufficient on their own to ensure active clinical utilization. Bridging the gap between structural existence and functional engagement requires institutional integration—specifically through accredited training, designated points of contact, internal ethics advisors, and an institutional culture of deliberation. For health systems internationally, these findings show that translating ethics governance into meaningful bedside practice depends on operational resourcing and clinical integration rather than legal mandate alone.

## Background

Healthcare providers routinely face complex ethical dilemmas intertwined with clinical decision-making. The Israeli Patient’s Rights Act (IPRA, 1996) legally framed the caregiver-patient relationship and embedded fundamental patient rights in statutory law. The Act mandated the establishment of statutory ethics committees (ECs) in all medical institutions [1].

Unlike most countries that rely on advisory consultation services including the U.S. [2, 3] and the U.K [4, 5] the Israeli statutory EC functions as a quasi-judicial body balancing autonomy, beneficence, and public interest, with the authority to issue binding decisions. Other European countries use a hybrid model, wherein EC’s are mandated by law, yet serve as advisory, with decision making authority left to caregiver [6, 7].

In Israel, committees are formally appointed, and re-appointed every four years, by the Ministry of Health’s (MOH’s) Director-General typically through an official letter defining membership and tenure – a process that underpins their legal authority. Committees must include a District Court-eligible chair (jurist), two specialist physicians, a psychologist or social worker, a registered nurse, and a public or religious representative- ensuring multidisciplinary deliberation across clinical, psychological, social, legal, and theological dimensions.

Most notably, the IPRA legally authorizes ECs to allow for deviation from the principle of patient autonomy in defined circumstances including providing treatment against the wishes of a competent patient, withholding information from a patient, disclosing medical information without consent to protect public health or a third party, and withholding or reporting genetic test results. In these legally defined contexts, the statutory EC functions as a quasi-judicial body with binding decision-making authority. This binding authority applies only when the committee operates within its statutory mandate in accordance with the law, and not in all cases brought before it. Prior to the IPRA, ECs functioned solely as institutional advisory bodies. The Act created a distinct statutory entity that many hospitals now also employ for broader ethical consultation. However, in this advisory role, committee recommendations are non-binding, meaning they do not carry legal force and serve only as professional guidance for clinicians and institutions.

The 2015 State Comptroller’s report revealed substantial gaps in the implementation of these legal entities, noting that only a minority of hospitals had established functioning ECs, and very few had convened in recent years. The report identified barriers to EC activity, including time constraints, heavy workload, difficulty distinguishing ethical from clinical dilemmas, limited ethics education, and member unavailability for urgent cases. The Comptroller urged the MOH, Health Maintenance Organizations (HMOs), and hospital leaders to analyze underuse of ECs, train EC members, and expand activity in line with international standards [8].

In addition, a national MOH survey, conducted the same year (2015), found that 70.9% of hospitals (78% of general hospitals and 50% of geriatric hospitals had statutory ECs; 25.5% operated separate advisory committees, and 20% shared a committee with another institution (here after- “shared committee”). Administrative compliance was partial: only 35.9% of institutions appointed committee substitute members and 66.7% produced written meeting protocols [9].

Against this backdrop, a multi-professional inter-ministerial committee was established to set binding standards, promote training, and monitor implementation of ECs. A major milestone was the publication of the 2018 Ministerial Guidelines, defining EC structure, procedures, and member training requirements [10].

The 2022–2023 national follow-up survey, described and analyzed in this paper, aimed to assess the structural, administrative, and operational implementation of ECs following the 2018 directive, examine institutional activity levels, and identify organizational factors and barriers associated with low committee utilization.

## Methods

This study is a secondary analysis of the national cross-sectional survey, conducted by the Israeli MOH in the years 2022–2023. The survey, designed by the Division of General Medicine for internal quality assessment, was distributed to all 73 institutions required by law to maintain a statutory EC: general, geriatric/rehabilitation, and psychiatric hospitals, MOH district bureaus, HMOs, and the IDF.

### Participants

Fifty-two institutions responded (response rate: 71%), representing all institutional types, ownership categories and geographic regions. A Fisher-Freeman-Halton exact test confirmed no significant differences between respondents and invitees by type, supporting representativeness.

### Instrument

A structured questionnaire, developed by a multidisciplinary MOH expert panel included both closed- and open-ended items across seven domains:

(1) EC structure (2) appointment and governance (3) administrative practices (4) visibility and accessibility (5) activity indicators (6) Training, and (7) barriers and improvement suggestions. Institutional variables (type, size, ownership, region) were added from the MOH database. Hospital size was categorized by number of beds as small (< 301 beds), medium (301–800 beds), and large (> 800 beds), (range: 58- 1636).

### Operational definitions

The following terms were used in this study as follows: A *Statutory EC* refers to a committee formally appointed under the IPRA with authority to issue legally binding decisions within its defined mandate. An *Advisory EC* refers to a non-statutory-consultative body providing ethical guidance. A *Joint EC* refers to a statutory committee that also performs advisory committee functions. A *Shared EC* refers to a single committee serving more than one institution. An *Application* was defined as a formal request submitted to the committee for deliberation and documentation, whether statutory or advisory. A *Meeting* was defined as a formally convened committee discussion, whether in person or virtual, documented in institutional records.

### Comparisons

Findings were compared with aggregated data from the 2015 national survey. EC activity was analyzed annually for 2016–2021, contrasting pre- and post-directive periods: 2016–2018 vs. 2019–2021. Activity was examined both continuously (mean number of meetings/applications per year) and categorically (low vs. high activity, with low activity defined as ≤ 1 meetings or applications per year). Notably, low EC activity was conservatively defined as a committee that reported both one or fewer (≤ 1) meetings AND one or fewer (≤ 1) applications per year. The committee was classified as having high activity if it exceeded this threshold in either parameter (i.e., > 1 meeting or > 1 formal application annually). This operational definition ensures that committees with substantial deliberation volume – whether driven by internally initiated meetings or external referrals handled without a formal convened plenary – were not erroneously classified as low-activity.

### Analysis

Data were analyzed using IBM SPSS Statistics v29. Descriptive statistics summarized structure and activity. Categorical associations used Chi-square or Fisher-Freeman-Halton exact tests; non-parametric continuous data used Mann-Whitney U test. Temporal comparisons applied McNemar and Wilcoxon signed-rank tests. Factors associated with low activity were examined using logistic regression and Generalized Estimating Equations (GEE) models to account for correlated observations within institutions. All tests were two-tailed (*p* < 0.05).

### Ethics

Data, collected for governmental quality control, were anonymized. The University of Haifa Research Ethics Committee approved this secondary analysis, approval number 365/24.

## Results

The results describe the current structure, governance, and activity of ECs in Israeli medical institutions, based on the 2022–2023 MOH survey, with comparisons to available data from 2015.

### Structural consolidation

Statutory ECs were present in almost all institutions, reflecting a marked expansion since 2015. Hospitals were nearly five times more likely to have a statutory EC in 2022 compared with 2015 (OR = 4.97, 95% CI 1.29–19.2, *p* = 0.020). As shown in Table 1, statutory ECs were most prevalent in general and psychiatric hospitals.

In 2022–2023, advisory activity was most often organized through joint statutory-advisory committees. Nearly two-thirds of institutions (63.5%) reported operating a joint committee, whereas one quarter (25.0%) maintained an independent advisory EC. Joint committee were especially common in general hospitals. Comparable data on joint committee structures were not collected in the 2015 survey. The prevalence of shared ECs was 15.4% in 2022–2023, compared with 20% in 2015. Internal ethics advisors were appointed in approximately one-third of institutions, exclusively in general hospitals.

**Table 1:** Distribution of statutory and advisory EC structures among responding institutions by institution type 2022–2023 (*N* = 52). Values are n (%)

Ethical Structures	General Hospitals ^a^(*n* = 30/38)	Geriatric/ Rehabilitation Hospitals ^a^(*n* = 9/12)	Psychiatric Hospitals ^a^(*n* = 6/12)	MOH Bureaus ^a^(*n* = 4/6)	HMO ^a^(*n* = 2/4)	IDF ^a^(*n* = 1/1)
Statutory EC	28 (93.3%)	7 (77.7%)	6 (100.0%)	3 (75.0%)	2(100.0%)	1 (100.0%)
Joint EC	22 (73.3%)	5 (55.5%)	2 (33.3%)	2 (50.0%)	1 (50.0%)	1 (100.0%)
Separate Advisory EC	10 (33.0%)	1 (11.1%)	1 (16.6%)	0 (0.0%)	1 (50.0%)	0 (0.0%)
Internal Ethics Advisor	10 (33.3%)	0 (0.0%)	0 (0.0%)	0 (0.0%)	0 (0.0%)	0 (0.0%)
Shared EC	5 (16.7%)	1 (11.1%)	0 (0.0%)	3 (75.0%)	0 (0.0%)	NA

^a^ Column headings indicate the number of responding institutions out of the total number invited in each institutional category; percentages within the table are calculated using the responding institutions as the denominator

### Administrative compliance

Administrative compliance indicators were higher in 2022 than in 2015 (Table 2). Most committees now operate with full statutory composition, written documentation of meetings, and formal summaries of decisions. However, documentation practices remain inconsistent, particularly with respect to recording committee decisions in patients’ medical charts and maintaining comprehensive archival storage.

Governance procedures also varied. Only about 81% of statutory ECs were formally appointed by the MOH Director-General, as required by law. Appointment rates were highest in general and psychiatric hospitals and lower in geriatric hospitals, district health bureaus, and public/NPO-owned institutions.

**Table 2:** Administrative practices of statutory ECs in medical institutions (*N* = 47)

Administrative practices	All Institutions with statutory EC
6 EC members	47 (100.0%)
6 EC members’ substitutes	39 (83.0%)
Written protocol every meeting	45 (95.7%)
Reasoned summary of EC decision	45 (95.7%)
Summary decision in medical chart	32 (68.1%)
Delivery of EC decision to applicant is documented	35 (74.5%)
Unlimited duration of storage	31 (66.0%)

Values are n (%)

### EC activity: meetings and applications

Meetings and applications represent related but not identical indicators of EC activity. EC meetings may be convened in response to formal applications, but also independently, for example to discuss recurring ethical challenges, institutional policies, or fundamental ethical issues. Conversely, not all applications necessarily result in a formal committee meeting. Accordingly, meeting frequency and application volume are reported separately in the analyses below, as they capture complementary aspects of EC activity. Compared with 2016–2018, statutory EC activity appeared higher in 2019–2021, as reflected in higher meeting frequency and a greater proportion of institutions meeting more than once per year (Table 3).

**Table 3:** Statutory EC meeting activity, 2016-18 vs. 2019-21 (*N* = 42)

Measure	2016–2018	2019–2021
Mean number of meetings per institution over the 3-year period (SD)	2.45 (3.43)	3.86 (4.48)
^a^ Sum of meetings across all institutions (3-year period)	103	162
Institutions with zero meetings	20 (47.6%)	13 (31.0%)
0–1 meetings/year (low activity)	33 (78.6%)	26 (61.9%)
> 1 meeting/year^b^	9 (21.4%)	16 (38.1%)

^a^Wilcoxon signed-rank test (*p* = 0.014)

^b^McNemar’s test (*p* = 0.016)

Values are mean (SD) or n (%)

Applications to statutory ECs were higher in 2019–2021 than in 2016–2018 across all legally defined categories: refusal of treatment, withholding information, and disclosure to third parties (Table 4). The largest difference was observed in disclosure-related cases, while treatment refusal remained the dominant topic overall.

**Table 4:** Applications to statutory EC by issue, 2016–2018 vs. 2019–2021

Application to statutory EC	Period	*N* institutions	institutions with ≥1 Application *n* (%)	Mean applications per institution (SD)	Median (range)	Total Applications (sum across institutions)
Withholding Information from Patients	2016–2018	41	10 (24.4)	0.44 (0.95)	0 (0–4)	18
	2019–2021	41	11 (26.8)	0.51 (1.34)	0 (0–6)	21
Refusal of Treatment	2016–2018	41	17 (41.5)	1.90 (3.77)	0 (0–20)	78
	2019–2021	42	22 (51.3)	2.14 (3.45)	1 (0–15)	90
Delivery of Information to 3rd Party	2016–2018	41	6 (14.6)	0.24 (0.69)	0 (0–3)	10
	2019–2021	41	11 (26.8)	0.29 (0.51)	0 (0–2)	12

Values are n (%), mean (SD), median (range)

Applications to statutory ECs accounted for nearly 60% of all submissions in 2016–2018 and 54% in 2019–2021. Although advisory ECs received fewer applications overall, their relative share was higher in 2019–2021, approaching half of all applications (Table 5).

**Table 5:** Applications to statutory and advisory ECs, 2016–2018 vs. 2019–2021

Application Type	Period	Mean annual applications per institution (SD)	Sum of Applications	% of Total Sum of Applications
Statutory (*N* = 40)	2016–2018	3.40 (4.49)	136	(59.9%)
Advisory (*N* = 39)^a^		2.33 (3.80)	91	(40.1%)
Statutory (*N* = 40)	2019–2021	3.53 (4.36)	141	(53.6%)
Advisory (*N* = 39)^a^		3.13 (4.63)	122	(46.4%)

^a^One institution did not report advisory EC applications, therefore *N* = 39 for advisory EC analyses

Values are n (%), mean (SD), median, and total (%)

Thematic analysis of applications revealed differences in the types of issues addressed across institutional settings. Treatment refusal remained the predominant issue in general hospitals; issues related to impaired decision-making capacity or guardian involvement were more frequent in geriatric institutions; and psychiatric hospitals addressed more dilemmas related to consent, confidentiality, and coercion. Broader themes included patient autonomy, guardianship, end-of-life care, confidentiality, fertility and IVF, research participation, and institutional policy.

### Advertisement and visibility

Almost 80% of institutions advertised their ECs to staff, whereas only 57.1% did so to patients and families. In the Northern District, all institutions advertised to staff and 80% to patients/families, with higher proportions compared with other MOH districts (p = 0.001 and p = 0.037 respectively) (Table 6).

**Table 6:** Advertisement of ECs by MOH district regions (*N* = 42)

Four Main MOH District Regions	Advertisement to Staff	Advertisement to Patients/Families
Northern District (*n* = 15)	**15 (100.0%)**	**12 (80.0%)**
Jerusalem District (*n* = 6)	2 (33.3%)	1 (16.7%)
Central District (*n* = 18)	14 (77.8%)	10 (55.6%)
Southern District (*n* = 3)	1 (33.3%)	1 (33.3%)
Total (*n* = 42)	32 (76.2%)	24 (57.1%)
^a^(Fisher-Freeman-Exact Test)	*p* = 0.001	*p* = 0.037

^a^p-values were based on Fisher’s Exact Test due to > 20% of expected counts < 5

Values are n (%)

Although regional sample sizes differed substantially- particularly in the Southern and Jerusalem districts- this comparison remains important for identifying geographic patterns in EC visibility and access. However, findings should be interpreted cautiously.

The presence of a designated contact person was associated with higher levels of advertising. Higher rates of advertising were observed in institutions with a contact person, both to staff (100% vs. 50%, *p* < 0.001) and to patients/families (100% vs. 0%, *p* = 0.017). Advertising methods varied: for staff, intranet postings were most common, followed by internal guidelines and email announcements; for patients and families, institutional websites and admission brochures were the primary channels.

### Training of EC members

Overall, 55% of committee members had not receive any ethics training. Nonetheless, 80% of respondents reported a need for a structured national training program. Training prevalence varied widely by institution type, ranging from complete absence in psychiatric hospitals to over 70% in geriatric/rehabilitation hospitals. Moderate training rates were observed in general hospitals (~ 52%), while training was less common in MOH district bureaus and HMOs/IDF (25% and 33%, respectively).

Regional differences in training prevalence were observed. Institutions in the Northern and Haifa districts reported higher training rates than those in other regions (10/14 [71.4%] vs. 11/32 [34.4%], χ²=5.771, *p* = 0.016), and similar regional patterns were observed for EC activity in the multivariable models (Tables 7 and 8). No significant association was observed between hospital size and training status.

Training formats included formal institutional programs, internal chair-led instruction, external expert-led workshops, and experience-based learning. Formal training typically consisted of structured courses or workshops organized by hospitals or external bodies, while chair-led training involved in-house guidance provided by EC chairs or senior members. External training was delivered through workshops or lectures led by ethics experts outside the institution. Experience-based learning relied on participation in committee deliberations and case discussions, often without a formal curriculum.

### Factors associated with low activity (≤ 1 meetings and application per year)

Multivariable analyses identified several factors associated with low EC activity, defined as ≤ 1 meeting or application per year (Tables 7 and 8). Smaller hospitals and geriatric facilities were associated with higher odds of low activity compared with large general hospitals. Private ownership was also associated with lower activity. Low activity was associated with absence of trained members and internal ethics advisors. Conversely, institutions with both trained members and a designated contact person were associated with lower odds of low activity.

**Table 7:** Factors associated with low EC meeting activity (medium/large hospitals only) (*n* = 24 paired)

Factors (reference)	Adjusted OR	95% CI	*P* value
Year: 2019-21 vs. 2016-18	0.37	0.17–0.80	0.012
MOH region: Group 2 vs. 1 (others vs. central)	4.54	0.85–24.36	0.078
Internal Ethics Advisor: Yes vs. No	1.02	0.18–5.72	0.983
Members trained: Yes vs. No	0.30	0.05–1.81	0.188

**Table 8:** Factors associated with low statutory-EC application activity (all hospitals; *n* = 34 paired)

Factors (reference)	Adjusted OR	95% CI^a^	*P* value
Year: 2019-21 vs. 2016-18	0.59	0.26–1.33	0.200
^b^Size: Medium vs. Small	0.17	0.03–1.11	0.064
^b^Size: Large vs. Small	0.02	0.001–0.32	0.006
MOH region: Group 2 vs. 1 (others vs. central)	3.09	0.80-11.92	0.101
Internal Ethics Advisor: Yes vs. No	10.38	0.73- 147.16	0.084
Members trained: Yes vs. No	0.86	0.22–3.41	0.832

^a^CIs are wide for some covariates due to sparse cells; interpret magnitudes cautiously

^b^Hospital size categories were defined by number of beds as small (< 301 beds), medium (301–800 beds), and large (> 800 beds)

## Discussion

In most countries across Europe, the U.S., and Australia, clinical ECs operate primarily in an advisory capacity, serving as forums for ethical reflection and deliberation rather than statutory bodies with legally binding authority [17, 20]. This purely consultative status frequently translates into substantial institutional heterogeneity across hospitals.

In the U.S., for instance, a 2017–2018 national survey revealed that while most general hospitals had established an EC, prevalence varied considerably by institution type, with lower adoption rates in small (< 100 beds), nonteaching, and rural facilities. Furthermore, although the median annual consultation volume per hospital remained unchanged at three compared to 2007, it had more than doubled in larger facilities (400 + beds). Nonetheless, among hospitals maintaining an EC, two-thirds conducted six or fewer consultations annually, and nearly three-quarters conducted 12 or fewer, exceeding nominal activity, yet reflecting relatively low utilization overall [18].

Similarly, in the U.K., the UK Clinical Ethics Network (UKCEN) identified 62 ECs, though activity levels remain uncertain and unmapped committees outside the network may exist. Comprehensive national data in the U.K. remain scarce due to the absence of statutory obligations requiring healthcare institutions to establish, resource, or maintain clinical ethics support services [19].

Conversely, in some European systems, such as Norway, the Ministry of Health legally mandates that all healthcare trusts maintain an EC, with the Centre for Medical Ethics (CME) at the University of Oslo providing national coordination and operational support. Consequently, core elements of committee structure and function are standardized, fostering greater institutional uniformity [21]. However, Norwegian ECs function strictly in an advisory capacity without binding decision-making authority; even when a case undergoes formal committee deliberation, ultimate decision-making responsibility resides solely with the treating clinician [22].

Unlike those international models, which operates exclusively or mostly in a consultative capacity, the Israeli framework institutionalizes legal authority alongside ethical deliberation, creating a distinctive interface between regulation and ethical reflection.

The 2018 MOH directive marked an important phase in the translation of statutory principles established in 1996 into operational standards. In the period following its publication, statutory EC coverage was higher across institutional sectors, consistent with convergence at the level of formal structure. At the same time, consolidation took different organizational forms across hospital types. General hospitals reached near-universal statutory coverage and more frequently integrated advisory activity within joint statutory-advisory committees, whereas geriatric and rehabilitation hospitals showed substantial but incomplete uptake, and smaller or community-based institutions more often relied on shared committee arrangements. These patterns may suggest that while a common statutory framework has been widely adopted, ethics functions are embedded differently within local governance structures. The relative lower prevalence of shared ethics committees in 2022–2023, compared with earlier data, alongside the higher prevalence of institution-based statutory committees, may reflect a shift toward intra-institutional models of ethical decision-making, particularly in larger hospitals. Nevertheless, shared committees remain a relevant structural option for smaller institutions, offering pooled expertise and continuity in settings with limited internal capacity.

Structural consolidation has not been uniformly accompanied by standardized administrative practices. Alongside improvements in administrative procedures, documentation and long-term record storage remained inconsistent, which may limit opportunities for institutional learning and accountability. International guidance identifies systematic documentation as a core standard of effective EC practice [11, 12]. Governance procedures also varied across institutions. Formal appointments confer legal standing and procedural legitimacy; its absence may be associated with weaker perceived authority and consistency. Administrative rigor may therefore be viewed not merely as a bureaucratic requirement, but as a structural condition associated with transparency, accountability, and public trust in ethical decision-making.

Beyond formal requirement, the findings suggest broader use of standardized procedures, documentation practices, and designated contact persons across diverse institutional settings, including general, geriatric, psychiatric and community-based institutions. These patterns are consistent with greater administrative alignment and the emergence of a shared ethical terminology across sectors.

However, structural consolidation was not consistently accompanied by sustained functional engagement. More than half of institutions reported one or no EC meetings per year, and several received no applications at all. Ethical deliberation structures are widely in place, yet they may not be consistently embedded as practical resources for ongoing clinical support and decision-making. At the same time, meeting frequency and application volume reflect related but distinct dimensions of EC activity and do not capture informal or preliminary ethics consultations that occur outside formal committee processes. When considered together, these findings suggest a gap between the formal establishment of ECs and their routine use in clinical practice.

Visibility of ECs’ activity differed substantially by target audience and institutional context. While most institutions reported advertising EC services to staff, substantially fewer provided comparable information to patients and families. Regional variation was also evident, with institutions in the Northern District reporting consistently higher levels of visibility. Taken together, these patterns constitute gaps in equitable access to the ethical decision-support mechanism within the framework of the Patient Rights Law (IPRA).

Notably, the presence of a designated contact person was strongly associated with higher levels of advertising to both staff and patients. This finding may suggest that visibility is linked to accessibility, as identifiable points of contact may translate abstract committee structures into usable institutional resources. In contrast, advertising alone, particularly when limited to passive channels, may be insufficient to support routine engagement with ethics services in the absence of clear referral pathways and operational support.

Training appears to be an important component of EC capacity. Despite widespread structural establishment, more than half of EC members had not received formal ethics training, and training provision varied substantially across institution types and formats. While some institutions reported structured institutional or external training programs, others relied primarily on chair-led instruction or experience-based learning, reflecting the absence of a standardized national framework for ethics education. This aligns with qualitative observations by Sperling et al. [13], which confirm the lack of mandatory training requirements while highlighting an expressed need for structured education. Notably, Sperling et al. also identified a potential barrier to implementation: senior clinicians often view their extensive clinical experience as a sufficient substitute for formal ethics training. This perception underscores the necessity for a recognized, tailored national program that respects clinical expertise while providing essential ethical and legal tools.

Differences in training prevalence were also evident across regions. Institutions in the Northern and Haifa districts reported higher proportions of trained EC members, with similar regional patterns observed for EC activity in the multivariable analyses (Tables 7 and 8). This co-occurrence may suggest an association between training and committee engagement. At the national level, however, the persistence of substantial training gaps, together with strong expressed demand for structured education, may indicate the need for coordinated approaches to ethics training to support equitable access to effective ethical deliberation across institutions and regions (as elaborated further in policy recommendations). This is strongly supported by Sperling et al. [13], whose findings reveal that hospital ethics committees currently operate in complete institutional isolation, with committee members explicitly calling for national forums to facilitate inter-institutional knowledge sharing.

Multivariable analyses further identified institutional characteristics associated with persistently low EC activity (Tables 7 and 8). Smaller hospitals and geriatric institutions were associated with higher odds of low meeting and application volumes compared with large general hospitals, which may reflect structural constraints in settings with limited organizational capacity. Private ownership was also associated with lower activity, which may reflect differences in governance priorities and institutional support for ethics deliberation.

In parallel, organizational support at the committee level was associated with activity patterns. Institutions lacking trained members or internal ethics advisors were more likely to report low activity, whereas committees with both trained members and designated contact persons were less likely to demonstrate inactivity. By contrast, advertising alone was not independently associated with increased activity, consistent with a distinction between visibility and functional accessibility. Together, these findings may suggest that sustained EC engagement is associated with the alignment of institutional capacity, professional expertise, and operational support rather than on formal structures or awareness measures alone.

The Israeli trajectory parallels global experience. In the United States (U.S.), over 95% of hospitals have ethics services, yet activity remains highly uneven across institutions. National surveys conducted two decades apart show that although the estimated total number of ethics consultations was higher in the later period, the median number of consultations per hospital has remained at 3, particularly in smaller and non-teaching hospitals. Higher consultation volumes were primarily observed in large teaching hospitals, while many institutions continue to report minimal routine use of ethics services [3, 14]. In the United Kingdom (U.K.), clinical ethics committees expanded to > 100 committees by 2010, yet activity remains uneven and may be associated with local leadership and institutional context [15]. Across Europe and Asia, ECs similarly struggle with limited utilization and variable clinician awareness [16, 17]. Israel thus shares a common global challenge of translating formal establishment of EC into meaningful engagement, despite its uniquely binding statutory framework.

The persistence of low activity levels may be related to organizational and cultural constraints. Frequently reported obstacles included the voluntary nature of EC participation, whereby committee work is typically performed in addition to regular clinical duties, without designated hours or formal institutional recognition. Such arrangements may limit member availability, continuity, and responsiveness. Similarly, committees lacking trained members, internal ethics advisors, or designated contact persons were significantly less active.

These challenges are not unique to Israel. National surveys of clinical ethics services in the U.K. describe variation in institutional support, training formats and integration into routine clinical practice [15].

The organizational vulnerability of ethics committees is further compounded by their unexamined relationship with hospital leadership. Although committee members frequently view their operational autonomy from hospital management as an essential safeguard for impartial deliberation, qualitative findings by Sperling et al. [13] indicate that this independence often reflects passive disconnect, administrative non-involvement, and a total absence of managerial oversight or performance evaluation. Sperling et al. note that hospital administrators regularly monitor other institutional committees through performance metrics and outcome reviews, whereas ethics committees are left in managerial isolation—a dynamic suggesting either leadership’s uncertainty regarding oversight or a broader failure to comprehend the strategic value of ethics committees. This disconnect directly aligns with our quantitative observations regarding low functional activity and limited organizational support across medical institutions.

Notably, the U.S. literature describes additional perceived barriers that were not reported by respondents in the present study. These include clinician perceptions of ethics consultation services as slow, difficult to access, or primarily relevant to high-stakes situations, such as end-of-life decisions, medical futility disputes, or guardianship conflicts, rather than as resources for everyday ethical dilemmas arising in routine clinical care [3]. The most recent U.S. national survey suggests that some structural barriers have persisted over time. Consultation activity remained concentrated in large teaching hospitals, while smaller institutions continued to report minimal routine use, which may reflect disparities in institutional capacity and resource allocation. In addition, the decline in formal evaluation of ethics consultation services suggests limited governance and feedback mechanisms, potentially limiting service development and visibility. Together with persistently unchanged levels of formal training, these findings may suggest that structural capacity, professionalization, and institutional support are key factors associated with ethics consultation activity [14].

These findings suggest that structural existence does not guarantee functional vitality. While Israel’s statutory framework has achieved broad institutionalization, sustained functionality requires embedding ethics consultation within the daily rhythms of clinical care. Awareness efforts and passive advertising also appear insufficient when not accompanied by accessibility, expertise and institutional support.

### Policy recommendations

The findings of this national survey highlight that the next stage in the evolution of Israel’s ECs must focus on functionality, competence, and equity rather than its mere structural establishment. While the MOH has established a robust regulatory foundation, sustaining and deepening EC activity now requires embedding ethics consultation into institutional culture, clinical workflow and professional incentive structures. Several interrelated policy directions emerge:

#### Establish a national training framework

Over half of the committees include members lacking official ethics training despite widespread recognition of the need for education in clinical ethics. A mandatory MOH-accredited program should define a consistent professional standard for EC membership. This framework could combine a national core curriculum- covering ethical theory, legal duties, deliberation methods, and documentation, alongside sector-specific modules tailored to general, geriatric, and psychiatric care. Periodic recertification and continuing education would ensure ongoing competence as ethical and legal contexts evolve.

#### Promote institutional integration and accessibility

Formal administrative backing, manifested through protected operational time, designated contact persons, electronic health record referral pathways, and inclusion of committee service in professional performance evaluations, is necessary to integrate ethics committees into routine institutional governance without compromising their ethical independence. Each hospital should appoint a designated contact person to serve as a focal point for referrals and liaison with the EC. Embedding EC referral options within electronic health-record systems, (e.g., “an Ethics Consult Order”) would normalize ethics consultation as part of routine care. Providing protected time and administrative support for EC members is essential to ensure availability, responsiveness, and sustainability of committee activity.

#### Formally recognized and rewarded ethics committee participation

Beyond protected time, meaningful incentives are required to sustain engagement in ethics training and committee work. Evidence from clinical ethics scholarship indicates that lack of recognition and career credit is a major barrier to participation in ethics consultation and committee service. Formal recognition of EC activity through professional evaluation frameworks, promotion criteria, academic credit, or modest financial remuneration signals that ethics work is valued institutional labor rather than voluntary extra work. Such recognition has been associated with improved recruitment, retention, and perceived legitimacy of ethics services [3, 18].

#### Develop regional mentorship and shared ethics support networks

Active centers could function as regional hubs, mentoring smaller and resource-limited institutions through joint training, shared case discussions, and cross-institutional learning. Such collaboration would reduce geographic and institutional disparities and strengthen capacity in geriatric and psychiatric settings as well as less active ECs in general hospitals.

#### Foster an ethical culture of deliberation and ethical voice

Structural mechanisms and visibility alone are insufficient to ensure meaningful engagement with ethics committees. Evidence from organizational research shows that ethical behavior is strongly shaped by institutional culture, including leadership role-modeling, openness to ethical discussion, and reinforcement of ethical conduct. Such environments and interventions promote ethical voice- the willingness of professionals to raise concerns and seek ethical guidance as part of routine practice [19, 20].

For example, a practical four-step training approach in hospital settings has been proposed, integrating ethics education into formal curricula, role-modeling by senior clinicians, clear institutional guidelines, and structured evaluation of ethical practice [21].

Accordingly, policy efforts should extend beyond awareness campaigns to deliberate cultural reinforcement. Leadership endorsement should explicitly frame ethical deliberation as integral to professionalism and clinical excellence. Integrating ethics discussions into departmental meetings, morbidity and mortality rounds, and continuing education can normalize ethical reflection in everyday clinical work. Recognizing participation in ethics consultation and committee activity through professional evaluation or promotion criteria would further reinforce ethics engagement as a valued component of clinical practice.

#### Establish a minimum national transparency standard for EC accessibility

This should include publicly available information on institutional websites, inclusion in admission materials, a clear referral pathway, and defined response times for urgent statutory cases.

Together, these strategies: training, integration, mentorship, monitoring, and cultural reinforcement, outline a coherent national policy pathway. They can transform Israel’s ECs from a legally mandated structure into a dynamic, learning system that strengthens ethical awareness, safeguards patients’ rights, build moral character and supports clinicians in complex moral decision-making.

### Limitations of the study

This study has several limitations. Its self-reported design may introduce recall and reporting biases. There may also be some variability in institutional reporting practices regarding key indicators such as “meeting” or “application” which could modestly affect comparability, as reporting practices were not fully standardized prior to the data collection.

Although the response rate was high and respondents were broadly representative of the invited institutions, non-respondents may differ systematically, limiting generalizability to smaller or less regulated facilities. Despite the use of a structured questionnaire, recall bias may have affected reports of meetings and applications across the six-year reference period (2016–2021). Social desirability Bias, in the form of over-reporting of compliance with Ministry of Health expectations, cannot also be excluded. In addition, the modest sample size- particularly within subgroups by type, size, or ownership, reduced statistical power for detecting certain associations. Also, while the study compared data from 2015 to 2022, not all institutions participated in both surveys, restricting longitudinal inference to system-wide rather than hospital-level trends.

In addition, the survey did not capture informal or curbside ethics consultations occurring outside formal committee structures. Consequently, unofficial, departmental, or bedside ethical deliberations were not assessed and cannot be quantified in this paper. Finally, the survey’s quantitative focus could not capture the qualitative dimensions and outcomes of ethical deliberation or contextual factors such as leadership culture and organizational support that may influence EC performance. It is worth noting that assessing the substantive ‘effectiveness’ of ethics committees, beyond meeting frequency and referral volume, remains conceptually and analytically challenging [22]. Standard quantitative process metrics cannot readily capture whether deliberations achieve communicative consensus [23], procedural consistency [24], or timely bedside support [25]. Future research is needed to develop multidimensional evaluative frameworks that examine how these institutional structures translate into meaningful ethical practice.”

## Conclusions

Israel’s experience reflects a distinctive legislative and organizational approach to the institutionalization of clinical ethics. The unique hybrid nature of the Israeli EC model, combining binding statutory powers with advisory functions, alongside detailed operational guidance issued over the past decade, led to a substantial expansion in the formal establishment of ECs across medical institutions. At the same time, findings from this national survey demonstrate that statutory mandates and top-down regulatory frameworks alone are insufficient to guarantee functional implementation and routine institutional utilization.

Globally speaking, the Israeli experience offers a key policy lesson: even within a robust, legally binding framework, structural consolidation does not automatically translate into functional engagement. Without internal institutional support, dedicated resources, and routine integration into clinical workflows, ethics committees risk remaining formal administrative entities rather than active, accessible consultative partners.

The structural consolidation of Israeli EC’s has not yet evolved into consistent functional engagement. The next phase in the evolution of ECs in Israel may therefore require a shift from existence to performance, from committees as formal entities to committees as active, trusted partners in clinical decision-making.

ECs thrive where expertise, accessibility, and institutional support intersect. These findings suggest that committees with trained members, internal ethics advisors, and clear referral pathways demonstrate substantially higher levels of activity. At the same time, limited visibility to patients and families indicates inequities in access to EC services within the IPRA framework. Building on this evidence, a coordinated national training framework, embedded within a broader policy of institutional integration, may help support the development of ECs into dynamic centers of ethical reflection and consultation.

Beyond technical and organizational measures, the consolidation of ECs also entails a cultural dimension. Embedding ethical deliberation into the everyday practices of healthcare may depend on the extent to which clinicians, managers, and patients perceive ECs as accessible, relevant, and supportive resources. Advancing this goal depends on visible leadership commitment, recognition of ethics related work as a legitimate professional contribution, and sustained collaboration among healthcare institutions, the MOH, and academic partners.

By strengthening training, integration, visibility and evaluative culture, Israel may further develop its statutory ethics framework in ways that support patient rights, assist healthcare professionals in addressing complex moral challenges, and contribute to public trust in the health system.

## Data Availability

Available from the corresponding author on reasonable request.

## Abbreviations

EC: Ethics Committee
IPRA: Israel’s Patients’ Rights Act
MOH: Ministry of Health
HMO: Health-maintenance organizations
IDF: Israel Defense Forces
U.S.: United States
U.K.: United Kingdom
